# Verdict against an Apothecary for an Alleged Mistake in Putting up a Prescription

**Published:** 1866-10

**Authors:** J. R. Lothrop


BUFFALO

### Medical and Surgical Journal.

VOL. VI. OCTOBER, 1866. No. 3.

### Original Communications.

ART. I.—*Verdict against an Apothecary for an alleged mistake in putting up a prescription.* BY J. R. LOTHROP, M. D.

The testimony of the witnesses for the plaintiff made out a case as follows: Mr. Farnell, a practicing lawyer of Lockport, N. Y., feeling somewhat ill, sent for Dr. Clark of the same city. This happened sometime in October, 1865. Dr. Clark prescribed in the case, as he alleged, the fluid extract of uva ursi., but when the prescription was sent to the drug store of Dr. Green, the clerk by mistake put in the fluid extract of veratrum viride instead, which being taken by the patient, caused alarming symptoms, followed by a serious illness of three or four weeks' duration, and such a serious impairment of health, as to lead to a suit for damages in the sum of $10,000. The case being tried at a term of the Supreme Court, Judge Grover, presiding; the result was, a verdict for the plaintiff for damages to the amount of $800.

In order to a full understanding of the case it will be necessary to give more of the details; and we cannot do better than to begin with the statement of Dr. Clark, the first witness for the plaintiff.

He stated, in effect, that being called in October, 1865, [he stated the exact date] to attend Mr. Farnell he found him suffering from a pain in the region of the bladder, *a difficulty in urinating*, and a slight swelling of the epididymis of one testicle. After due reflection upon the case he thought best to make the following

℞ Fl. Ext. Uva Ursi ℥ss.
Syr. simplicis ℥jjss.

writing also on the same paper, for several morphine powders of (I think) $\frac{1}{4}$ of a grain each. He then left, (having probably first given directions about the use and dose of the above medicine, as none were written upon the prescription, though I do not remember that he so stated.) About midnight he was summoned to attend Mr. Farnell. Arriving at the house he found another physician, Dr. McCollum, already there, and the patient in a condition which excited in him great alarm. He was pulseless, covered with a profuse cold sweat, breathing with difficulty, unable to speak because the attempt immediately brought on retching, beat ing his breast, and withal exhibiting a death-like pallor of face. Without stopping to direct measures for the sick man's relief, or rather leaving that matter to Dr. McCollum, who had very properly given stimulants, he at once seized the bottle containing the medicine, to which the patient by signs called his attention, and with that and the prescription ran as quickly as possible to the drug store. There he ascertained that veratrum viride was used in the prescription put up and sent to Mr. Farnell. He then went quickly to the house of the patient again, knowing the cause of the alarming trouble and prepared to act properly. He found time, however, in his journey to and from the drug store, to taste the medicine and *experience its emetic action on the way.* By a free use of stimulants, both by mouth and rectum, the patient rallied from his alarming prostration, and the doctor left him in the early morning, restored and comfortable.

Sometime in the forenoon of the same day he was again sent for and found his patient suffering from great abdominal pain, tenesmus and frequent discharges of blood and mucus, in fact he found all the symptoms of dysentery. This he treated energetically, by enemas of starch and laudanum, and after a few days there was some improvement. In fine, the dysenteric trouble continued quite severely for several days, and in the whole lasted about three weeks, keeping his patient confined to the house all that time, and nearly all that time in bed. During this dysenteric attack there were not only frequent discharges of bloody mucus, but portions of mucous membrane, so that, as he stated, complete casts apparently of the mucous membrane of the small intestines were thrown out and seen by him in the vessel. In order to illustrate this, the doctor rolled a piece of paper in the form of a cylinder and exhibited it to the jury. When his patient recovered from his attack of inflammation of the bowels he ceased attendance, and had never since been called to attend him professionally.

It will be observed above that the condition of the patient when Dr. Clark was first called to attend him, is but poorly described. When asked what the urinary trouble was he said he did not know, and could not state it to the jury. The matter is left rather indefinite, as to whether there was much disturbance or not; whether any excitement of pulse or not. The medicine intended to be given, viz: uva ursi, the doctor confessed was merely a placebo. On the prescription produced in court as the original one, no directions as to dose were written, but the dose of the mixture taken was one teaspoonful, which would contain about 10 or 15 drops of the fluid extract of uva ursi. When asked if such a dose would not be inert, the doctor replied that he supposed it would be almost wholly so, but that he never gave his patients strong medicine when he did not know what ailed them. A very honest confession; but it would have been much more to his credit to have confessed that under such circumstances he gave none; though we ought, in justice, to say that his practice in this respect was not so unusual as to be termed singular.

Dr. McCollum's testimony was confirmatory as to the condition of the patient, and his rallying under the use of stimulants. He seemed evidently to suppose the effects would only be temporary, and was not prepared to expect the serious trouble which was alleged to have followed.

The plaintiff himself being called to the stand gave in effect this statement. He had for some time previously been afflicted at times with a urinary trouble which occasionally made him unfit for his ordinary work. (As Dr. Clark could not state with accuracy what it was, of course he could not be more definite.) At the time mentioned, viz: October, 1865, being more than ordinarily troubled, he thought best to begin treatment, meaning to continue it. Accordingly he called in Dr. Clark, who left a prescription.

The medicine prescribed, as he supposed, was obtained from the apothecary's early in the evening, and he took of it one teaspoonful. About three hours after taking it, feeling rather peculiar sensations, nausea among them, he took whisky with sensible relief. Four hours after the first, he took another teapsoonful of this medicine. This was soon followed by most uncomfortable symptoms, among which were severe vomiting and retching and great distress about the precordia. He thought his breath was going, and he lost the power of speech; in fact, felt as if dying. Physicians were summoned, and soon Dr. McCollum arrived, followed not long after by Dr. Clark. He experienced relief from the measures taken by the physicians. Sometime in the forenoon following he began to experience intense, burning pain in the umbilical region, so intense that he could only express it by the term, agony. This was soon followed by discharges of bloody mucus, these also a source of great pain. For three weeks after this he suffered, more or less, most of the time, from pain and dysenteric discharges. He was under the impression that his health had never been fully restored. Before taking the medicine, with the exception of the trouble above stated, which he considered almost nothing, he had been a man of firm health, competent for all the demands of his laborious profession. Since then he had never been well; had occasional pains in the head unfitting him for work, was unable to endure fatigue, was easily exhausted by labor in the courts, especially felt great weakness and trembling in the limbs upon going up stairs. Had been obliged to refuse legal business.

It should be stated that plaintiff had the appearance of a man in sound health, and confessed that he was more fleshy than he had ever been, though he thought he weighed less than usual, i. e. that he was larger but not heavier. He had not, however, brought his notion to test of the scales. He had, moreover, never thought himself sick enough to need the services of a physician, i. e. after getting up from the first illness. We therefore have nothing but the statement of the plaintiff alone as to his condition for nearly a year. In such a case, it is evident that a large allowance must be made for the workings of the imagination. It would hardly be safe to admit that a man can judge correctly of his own case, especially when the departure from health is very slight, and the mind is prepared to exaggerate symptoms, if not actually to create, because expecting them. In order to show that the particular symptoms which he attributed to the medicine, were real after effects, and not imaginary, several rather robust looking men, who had taken large doses of veratrum viride, were put upon the stand. They stated that since taking the medicine they had not been in as good health as before, and probably had not expected to be. One had been, since, subject to a bad feeling in the head and shortness of breath; one felt weakness of limbs upon going up stairs; and though probably it was not very plain to many, yet they were firmly impressed with the idea that in some way health had suffered.

From the above imperfect statement some notion may be had of the alleged facts of the case as presented by the plaintiff. On the part of the defense the only positive testimony introduced, was that of the clerk who put up the prescription. He stated that he did not make a mistake, but that the prescription was written for veratrum viride, and that therefore the mistake, if one was made, was not his, but that of the physician who wrote it. The original prescription was taken away with the medicine it called for, and no copy made; so that the apothecary had no written evidence of the correctness of his clerk's statement. The original prescription was produced in court and sworn to by Dr. Clark. The defense endeavored to raise some question as to its being the same. They thought that there could be detected upon the prescription marks of alteration, as far as the name of the principal medicinal ingredient was concerned, but they could establish nothing. As far as the positive evidence was concerned, the mistake was that of the apothecary. The question of alteration was one of opinion merely, and it was raised not from mere caprice, but from appearances which made it proper enough.

Admitting then that the clerk made the mistake, about which, I suppose, the jury had not a particle of doubt, the question then arises, was the severe illness which the patient stated followed the taking of the medicine, directly caused by it? The question might be made even broader and include also peril to life. It seems very probable that Dr. Clark thought life endangered. Dr. McCollum was much alarmed at the condition, but when he understood the cause, as he soon did, his alarm quickly disappeared. The question of peril to life was only important in its relation to damages, as a medical question it ceased to have any point when the patient rallied. He did not die from its effects, therefore it would be profitless to discuss what it might have done. But undoubtedly a jury would be influenced in estimating the injury to health by the idea that the peril to life was great. Therefore the general question of the liability of veratrum viride to destroy life was gone into.

Before going farther it will be well to try to arrive at some idea of the quantity of veratrum viride taken. The mixture contained a half an ounce of Thayer's fluid extract of veratrum viride in two and a half ounces of syrup. The proportion of veratrum viride then was one-sixth. Calling a teaspoonful 70 or at most 80 drops, 12 or 13 drops of the medicine would be contained in it. Two teaspoonfuls were taken at an interval of four hours, consequently two doses of 12 or 13 drops were taken. Would such a quantity, in the first place, destroy or peril life? and secondly, would it be likely to cause an attack of dysentery? Upon these two points the defense introduced the testimony of a number of physicians, the writer among them. Drs. Gould, Babbitt, Foote and Leonard, physicians of Lockport, men of ability and well qualified by reading and experience, to give opinions upon the subject, were also of the number. The medical witnesses all concurred in the opinion that in pretty large doses, veratrum viride could not be considered dangerous to life; and in the second place, that even a cathartic action following its administration was exceptional, so much so, that they should be extremely incredulous of any statements as to such an effect.

As to the possibility of its proving such an irritant to the bowels as to cause an attack of dysentery, difficult as it is for a medical man, when closely pressed, to say what *might or might not* happen, they were unwilling to admit the possibility of such an event. Several of the medical gentlemen had given the medicine in doses nearly as large, more frequently and for a longer time, so that the whole quantity taken would be much greater, without causing dangerous symptoms. I suspect the experience of many physicians has been the same. That, at most, twenty-six drops of veratrum viride (as medicine is ordinarily taken the probability is that not more than 20 drops in all were taken) taken in two equal doses, four hours apart, should give rise to such an amount of inflammation of the intestines, as to cause dysenteric discharges for several weeks, is so highly improbable, that most medical men would be almost ready to deny its possibility. We may be somewhat skeptical as to the statements made relative to some of the facts, or supposed facts, observed, or thought to have been observed; when we recall the statement made by the physician in attendance that, casts of the mucous membrane were thrown off.— It would hardly coincide with ordinary medical experience, that an inflammation so intense as to destroy whole rings of the mucous membrane of the intestine, should terminate in three weeks *in recovery;* and it would be still more anomalous that such serious disease should exist unaccompanied by discharges of pus. Moreover, tenesmus and discharges of bloody mucus are ordinarily taken as indications that the inflammation is located in the rectum and colon, not in the small intestine, whence the rings were thought to have come. These evidences of want of accuracy of observation in some respects, go to weaken confidence in the accuracy of observation and statement in most others. A man who could shed his intestinal mucous coat and escape ulceration and long continued trouble, even if he did not die, would certainly be an exception to ordinary humanity. Men, now and then, are accused of having rather thick skins, but if we rely upon these observations, it follows that a very tough mucous membrane may exist also. If the statements brought out by this case are reliable, our medical records will become possessed of something heretofore unprecedented, and Dr. Stillé must gather it in to be cited as authority, should another similar trial take place.

But if we admit that the serious events followed so soon upon the taking of the doses of veratrum viride above named, are they to be considered as consequences? It is not safe in medicine to argue *post hoc*, therefore, *propter hoc*. Can any theory of the case account for the alleged facts related? The theory of the case adopted by the physicians of Lockport, will most certainly and plausibly; it being only necessary to suppose that the condition of the plaintiff was not observed quite as carefully as it should have been. The theory was this: at the time the medicine was taken by mistake dysentery was prevailing. The pain spoken of as being felt in the lower part of the abdomen, not probably being very accurately referred, and supposed to be in the bladder, was really in the intestines, the sigmoid flexure or rectum for instance. Irritability of the bladder or painful micturition is not a very rare symptom in troubles of the rectum, in fact is observed in dysentery. These were the premonitory symptoms of dysentery. Perhaps a careful observation of the pulse and tongue would have given additional evidence. These appear to have been wanting. But there is evidence that morphine was provided for some purpose; and is a sort of evidence that it was provided for a pain, greater than we are led to believe existed by the statements in evidence. We are left in the dark, as to what use was to be made of the morphine, nothing was said about it. Of course, we can understand that morphine would be resorted to by a physician in conditions of pain, even when not very severe. But the testimony does not give us the idea that the *pain* was a noteworthy symptom.

As the case appears in evidence, it is simply this, a physician is called to treat a chronic urinary trouble, not of much importance, and making so little impression upon him that he prescribes a placebo, taking care, however, to have a medicine of real potency on hand. He orders the inert medicine to be taken once in four hours, and waits like Mr. Micawber for something to turn up. By mistake the so-called sick man gets a dose of a powerful drug, the immediate effects of which alarm all about him, but though not proving as dangerous as they appear, they are followed by a real sickness, lasting three weeks. The resulting sickness is charged upon the medicine, and this last is the point of the case. Now from the well known action of the drug such a result must, by all medical men, be pronounced extremely exceptional. On the other hand, it is not at all improbable that the same sickness would have happened, had no medicine been taken; taking into account the prevalence of dysentery at the time, and the similarity in most respects, excepting always the casts of mucous membrane, to a case of dysentery. Dysentery is not always preceded by serious disturbance, it may occur without very marked premonitory symptoms. Moreover, pain low down in the pelvis and difficulty in micturition, are probable, early symptoms. We need not presuppose in all cases great antecedent general disturbances, for it is often absent, but it might have existed in some degree and escaped detection by the physician. Now one point we wish to present is, that there is quite as great a probability that the physician, Dr. Clark, was mistaken as to the condition of his patient and failed to foresee what was coming, as that a medicine should act in a manner, according to most authorities, extremely exceptional. Another point is, that even if no premonitory symptoms existed, therefore, that there was no failure on the part of Dr. Clark of careful observation, the probability is as great that the medicine had nothing to do with causing the dysentery, as that it caused it. It might be expected that a jury would connect the giving of a medicine and events closely following in the relation of cause and effect, but medical men know well enough that fallacies attend this method of reasoning.

Though the case, therefore, makes a legal precedent, it need not necessarily be looked upon and quoted as a medical one, and we might say ought not to be.

Several questions arise as to the actions of veratrum viride and the teachings upon the subject, which we will reserve for another article.

